# Uncovering the Understanding of the Concept of Patient Similarity in Cancer Research and Treatment: Scoping Review

**DOI:** 10.2196/71906

**Published:** 2025-08-18

**Authors:** Iryna Manuilova, Jan Bossenz, Annemarie Bianka Weise, Dominik Boehm, Marvin Döbel, Silke D Werle, Arsenij Ustjanzew, Niklas Reimer, Cosima Strantz, Philipp Unberath, Patrick Metzger, Thomas Pauli, Susann Schulze, Sonja Hiemer, Irmak Oguztürk, Leila Kamkar, Hans A Kestler, Hauke Busch, Benedikt Brors, Jan Christoph

**Affiliations:** 1 Junior Research Group (Bio-)Medical Data Science Faculty of Medicine Martin Luther University Halle-Wittenberg Halle (Saale) Germany; 2 Medical Center for Information and Communication Technology Universitätsklinikum Erlangen Friedrich-Alexander-Universität Erlangen-Nürnberg Erlangen Germany; 3 Bavarian Cancer Research Center (Bayerisches Zentrum für Krebsforschung) Erlangen Germany; 4 Institute of Medical Systems Biology Ulm University Ulm Germany; 5 Institute of Medical Biostatistics, Epidemiology and Informatics (IMBEI), University Medical Center of the Johannes Gutenberg-University Mainz Mainz Germany; 6 Medical Systems Biology Group, Lübeck Institute of Experimental Dermatology, Universität zu Lübeck Lübeck Germany; 7 University Cancer Center Schleswig-Holstein, University Hospital Schleswig-Holstein Lübeck Germany; 8 Medical Data Integration Center, University Hospital Schleswig-Holstein Lübeck Germany; 9 Medical Informatics, Friedrich-Alexander-Universität Erlangen-Nürnberg Erlangen Germany; 10 SRH Fürth University of Applied Sciences Fürth Germany; 11 Institute of Medical Bioinformatics and Systems Medicine, Medical Center-University of Freiburg, Faculty of Medicine, University of Freiburg Freiburg Germany; 12 German Cancer Research Center (DKFZ) Heidelberg, Clinical Trial Office Heidelberg Germany; 13 Krukenberg Cancer Center Halle (Saale) Halle (Saale) Germany; 14 Department of Translational Medical Oncology, National Center for Tumor Diseases (NCT) Heidelberg and German Cancer Research Center (DKFZ) Heidelberg Germany; 15 Division Applied Bioinformatics, German Cancer Research Center (DKFZ) Heidelberg Germany; 16 German Cancer Consortium Heidelberg Germany; 17 National Center for Tumor Diseases (NCT) Heidelberg Germany; 18 Medical Faculty Heidelberg and Faculty of Biosciences, Heidelberg University Heidelberg Germany; 19 Data Integration Centre of the University Hospital Halle (Saale) Halle (Saale) Germany

**Keywords:** cancer research, patient similarity, precision oncology, cancer similarity metrics, scoping review

## Abstract

**Background:**

Patient similarity is a fundamental concept in precision oncology, offering a pathway to personalized medicine by identifying patterns and shared characteristics among patients. This concept enables stratification into clinically meaningful subgroups, prediction of treatment responses, and the tailoring of therapeutic interventions to individual needs. Despite its transformative potential, the definition, measurement, and clinical application of patient similarity remain inconsistently established, creating challenges in its integration into cancer research and clinical practice.

**Objective:**

This study aimed to synthesize evidence on the multidimensional concept of patient similarity in cancer research by analyzing its application across different points of possible data types, methodological frameworks, biological contexts, and commonly studied cancer types.

**Methods:**

This scoping review followed the PRISMA-ScR (Preferred Reporting Items for Systematic Reviews and Meta-Analyses Extension for Scoping Reviews) framework and the Joanna Briggs Institute guidelines. A systematic search was conducted across PubMed, MEDLINE, LIVIVO, and Web of Science (covering the period from 1998 to February 2024) and was supplemented by snowball sampling and manual searches. Duplicate records were removed, and study selection was carried out in 3 phases: title and abstract screening, disagreement resolution, and full-text screening. Each step was independently performed by 2 reviewers in Rayyan, with conflicts resolved by a third reviewer. Data extraction was performed using a predefined template to capture methodological approaches, data types, cancer types, and research objectives related to similarity in patients with cancer.

**Results:**

This scoping review synthesized evidence from 137 studies, emphasizing the multidimensional concept of patient similarity in cancer research, which integrates diverse data types, methodological frameworks, research objectives, and cancer types. Transcriptomic data (92/137, 67.1%) and clinical data (65/137, 47.4%) were the most frequently used, often combined to enhance the comprehensiveness of similarity analyses. Machine learning (76/137, 55.5%) and network-based approaches (72/137, 52.5%) were prominent methods, reflecting their capacity to handle complex, high-dimensional data and uncover intricate relationships. Cancer subtype identification (70/137, 51.1%) and biomarker discovery (41/137, 29.9%) were the primary research objectives, underscoring the centrality of patient similarity in precision oncology. Breast, lung, and brain cancers were the most frequently studied, benefiting from established research frameworks and abundant datasets. Conversely, rare cancers were underrepresented, highlighting a critical gap in the generalizability of current methodologies.

**Conclusions:**

This comprehensive scoping review examines the concept of patient similarity in cancer research and highlights the critical role of a multilayered perspective in capturing its complexity and identification to enhance understanding and application in precision oncology.

## Introduction

### Background

Precision oncology has fundamentally transformed cancer research and treatment by adopting personalized genetic, molecular, and clinical approaches for individual patients [1,2]. A key principle of this paradigm is patient similarity, defined as the identification of patterns and commonalities among patients based on tumor genomics, disease progression, and therapeutic response [3]. Recognizing biologically similar patients enables clinicians to select targeted therapies more precisely, improving treatment outcomes and minimizing adverse effects [4,5].

The idea of learning from similarities between patients has deep historical roots. Since the time of Hippocrates, who systematically observed symptom patterns to guide diagnosis and treatment, comparing similar cases has been a cornerstone of medical practice [6]. Over the centuries, this principle evolved from purely clinical observations to sophisticated molecular and genomic analyses, ultimately forming the foundation of modern precision medicine [7,8].

In oncology, the concept of patient similarity expanded notably in the 1970s and 1980s, when researchers began classifying cancers into subtypes based on histopathological features. This approach recognized significant heterogeneity in tumor behavior, prognosis, and treatment responses even within cancers originating from the same tissue [2,3]. The 1990s marked a turning point with the advent of molecular profiling techniques, such as gene expression analysis, which allowed for more precise subtyping and highlighted the relevance of molecular similarity in guiding therapeutic strategies [9].

The clinical utility of molecular-based patient similarity was exemplified by the development of targeted therapies. Landmark successes, such as trastuzumab for HER2-positive breast cancer and imatinib for chronic myeloid leukemia, demonstrated that patients sharing specific genetic profiles could benefit from tailored treatments [10,11]. These advances firmly established the importance of integrating individual molecular characteristics into treatment planning, laying the groundwork for personalized oncology [4,12]. More recently, large-scale genomic initiatives such as The Cancer Genome Atlas and the International Cancer Genome Consortium have enabled comprehensive comparisons of genetic and molecular profiles across diverse patient populations [13]. Progress in bioinformatics and computational methods throughout the 2010s further facilitated detailed analyses of multidimensional biological data, including genetic mutations, epigenetic modifications, gene expression patterns, and features of the tumor microenvironment [14-17]. These technological advances have greatly enhanced our ability to stratify patients based on complex molecular characteristics. However, despite these achievements, consistently defining and applying patient similarity in research and clinical practice remains a major challenge. Current approaches often rely on heterogeneous criteria, ranging from specific genetic mutations to broader clinical phenotypes, leading to inconsistent findings and complicating the translation of results into clinical decision-making [18]. In addition, the integration of diverse data types (eg, genomic, clinical, and imaging) poses significant technical and methodological difficulties [14]. Furthermore, the lack of consensus on how to determine patient similarity, due to the use of varying algorithms and models, leads to inconsistent findings [15]. Moreover, the inherent heterogeneity of cancer, including variations within the same tumor type, makes it even more difficult to identify similar patients [19].

To address these challenges, further research is needed to develop standardized metrics and methodologies for assessing patient similarity, which could open crucial research opportunities in precision cancer care. To underline this need and to provide a current understanding and application of patient similarity in the context of cancer, a scoping review was conducted. This review aims to offer a comprehensive understanding of the current applications and methodologies of patient similarity in cancer research, with the hypothesis that a multilayered perspective is essential for enhancing precision oncology and improving patient outcomes.

### Research Concept

This scoping review focuses on the concept of patient similarity in oncology across several interrelated aspects. It investigates methods and approaches for identifying and analyzing patient similarities by exploring diverse data types, such as genetic, molecular, and clinical information, to uncover patterns among patients with cancer. The review highlights the most commonly studied cancer types, identifies research trends, and pinpoints overlooked areas requiring further exploration. Despite growing interest in patient similarity, no prior scoping review has systematically examined both the qualitative and quantitative dimensions of this concept. While individual studies propose various definitions and methodological approaches, a comprehensive overview of these perspectives is lacking. Furthermore, the field lacks a synthesis of the most frequently investigated aspects of patient similarity, such as commonly used data types, analytical techniques, and dominant research trends. This review addresses these gaps by providing an integrated analysis of how patient similarity is defined, measured, and applied in oncology, offering insights that may enhance its clinical utility.

## Methods

### Overview

This scoping review was conducted in accordance with the PRISMA-ScR (Preferred Reporting Items for Systematic Reviews and Meta-Analyses Extension for Scoping Reviews) checklist and the Joanna Briggs Institute reviewer’s manual [20,21], with all steps guided by a preestablished protocol [22]. These provided a structured framework that ensured transparency and consistency throughout the review.

### Inclusion and Exclusion Criteria

Our primary goal was to include a broad range of studies to provide a comprehensive overview of research on similarities in patients with cancer. To ensure quality and relevance, we established specific inclusion and exclusion criteria as predefined in our protocol.

Studies were included if they provided substantial evidence on similarities in patients with cancer across diverse populations, ages, genders, and cancer types. We limited the review to studies published within the last 25 years and written in English or German, the working languages of our team, to facilitate thorough analysis and to focus on contemporary developments relevant to current cancer research. Studies were excluded if they did not address similarities among patients with cancer, were bachelor’s or master’s theses, were unpublished manuscripts, or focused on noncancer conditions or animals. Pilot testing on a sample of studies helped refine the criteria, enabling the selection of the most relevant studies for our scoping review.

### Applied Search Strategies

Our search strategy used a triangulated approach, combining keyword searches, snowball sampling, and manual review for a comprehensive literature assessment. Details regarding the search strategy, including the specific search queries for the databases, are outlined in the protocol [22]. Final searches were completed on February 7, 2024.

### Study Selection Process

The selection process followed a rigorous 3-stage approach—title-abstract screening, disagreement resolution, and full-text screening—aligned with the recommendations by Tricco et al [20]. To ensure quality, each stage included pilot testing with calibrated forms for standardization [21,22]. Publications were independently reviewed by at least 2 blinded reviewers using predefined eligibility criteria. Disagreements were resolved by an independent reviewer, with all discrepancies documented for transparency [21]. Screening was conducted using Rayyan (Rayyan Systems Inc), a web-based software [23], and outcomes were summarized using the PRISMA-ScR flowchart.

### Evidence Charting

In this scoping review, the research team manually extracted data using a predefined, pretested template designed to align with the research questions. This approach ensured systematic data capture, consistency across studies, and minimized errors. Pilot testing confirmed the template’s robustness and accuracy, and the original templates are available in the protocol without modifications.

### Data Analysis

The analysis process followed a structured series of standardized steps, including data preprocessing, categorization, methodological classification, and quantitative and qualitative analyses. All data generated or analyzed during this study are included in this paper and Multimedia Appendix 1 [15,18,24-158].

#### Data Preprocessing

The data preprocessing aimed to establish a unified dataset for systematic analysis. Extracted data were rigorously standardized to ensure comparability across sources, with consistency and completeness checks addressing any inaccuracies by referencing the original sources. The terminology, metrics, and scales were aligned to minimize discrepancies and facilitate seamless integration.

#### Data Categorization

A structured categorization of the reviewed literature was conducted to enable quantitative analysis (Multimedia Appendix 2). Each paper was systematically analyzed and assigned to categories based on the research questions. In this process, papers were first assigned to initial categories, which were subsequently merged into overarching categories representing the resulting classification for the final quantitative analysis. Specifically, the papers were categorized by their contribution to defining patient similarity, methods for analyzing patient similarity, data types used, primary tumor location, and cancer types. This framework provided a comprehensive basis for identifying patterns and trends across studies.

To obtain an overview of the exact purpose of the selected publications, they were assigned different main objectives. Only the stated primary objectives of the studies were considered. For example, many studies that focus on subtype identification also automatically identify biomarkers that are relevant to this subdivision. Nevertheless, these studies were assigned to the subtype identification category only if this was specified as an objective. However, it was possible for publications to be assigned to several destinations if required. To identify which methods are particularly important for determining patient similarity, the methods used were assigned to each paper. Only methods crucial to the publication’s content were considered. They were grouped into three main categories: (1) machine and deep learning approaches, (2) network- and graph-based approaches, and (3) statistical and advanced mathematical techniques. A publication was assigned to a category if at least 1 main method of the work could be assigned to this category. In cases where studies incorporated a combination of methodological approaches, they were assigned to multiple categories.

Machine and deep learning approaches were categorized separately because of their automated learning capabilities, ability to handle large-scale datasets, and suitability for predictive and classification tasks in cancer research. Network- and graph-based approaches were grouped independently because of their emphasis on relationships and interactions between biological entities, such as genes, proteins, or patients. Visualizing data as graphs or networks provides intuitive insights into connectivity patterns, making these methods particularly effective for exploring complex interactions involved in cancer biology. Statistical and advanced mathematical techniques were classified separately for their theoretical nature and their role in validating findings from computational methods. This categorization offers a comprehensive overview of methodologies in patient similarity literature and highlights overlaps between approaches.

To enable a comprehensive analysis of the data types, an overarching categorization was established. As specific data types alone did not allow for significant statistical analysis, 6 main categories based on data types mentioned in scientific papers were introduced: clinical data, genetic and genomic data, transcriptomic data, epigenetic data, proteomic and metabolomic data, and pathway and network data. This categorization facilitates more lucid and interpretable analysis results, with each paper assigned to multiple data categories as required. Analyzing cancer types required classifying and subdividing specific cancer types into supergroups. Using OncoTree (Memorial Sloan Kettering Cancer Center) [159] as a basis, primary tumor sites were initially categorized, followed by expert review and minor adjustments. Publications often investigated multiple tumor sites or cancer types, and their classification reflects this complexity. The Results section provides a detailed overview of these categorizations.

#### Qualitative and Quantitative Analysis

This analysis used both quantitative and qualitative methods to examine the dataset. Quantitative analysis assessed frequency and recurring themes, identifying similarities and differences to clarify trends. In this part of the analysis, all publications were given equal weight. Qualitative analysis synthesized diverse perspectives, deriving nuanced insights and coherent conclusions. Together, these approaches ensured a well-rounded understanding of the dataset by aligning quantitative and qualitative insights.

## Results

### Summary of General Findings

**Figure 1:**
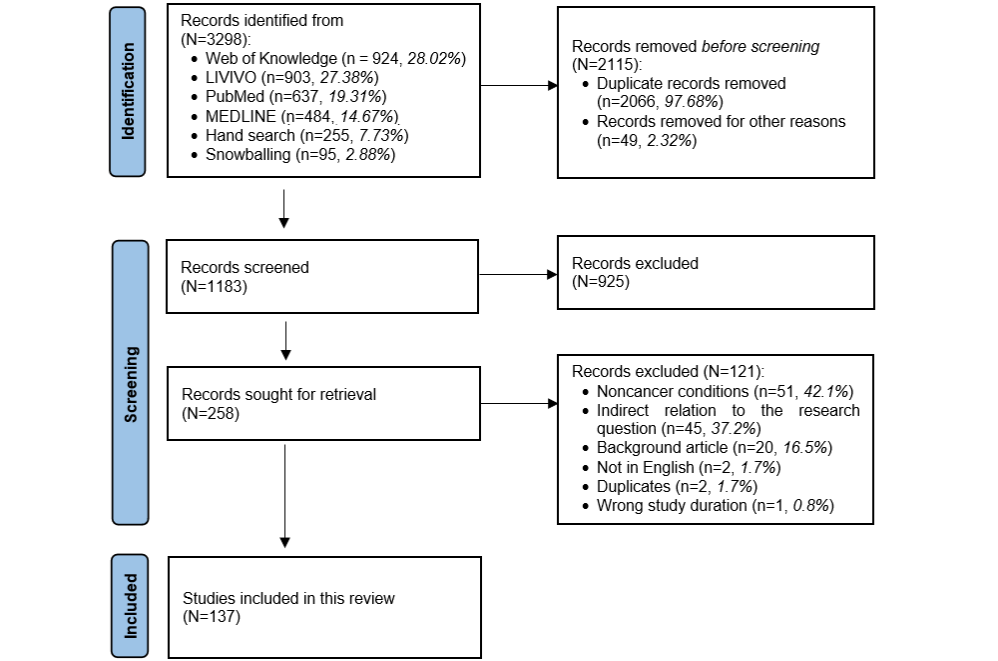
PRISMA (Preferred Reporting Items for Systematic Reviews and Meta-Analyses) flow diagram of the review process. The process comprised 3 main phases: identification of records through database searching and additional sources, screening of titles and abstracts, and full-text assessment for eligibility. After applying the predefined inclusion and exclusion criteria, 137 studies were included in the final review.

Figure 1 illustrates the PRISMA (Preferred Reporting Items for Systematic Reviews and Meta-Analyses) flowchart [20], detailing the process of narrowing down 3298 initially identified records to the 137 studies ultimately included in the review. Records were sourced from PubMed (637/3298, 19.31%), MEDLINE (484/3298, 14.67%), LIVIVO (903/3298, 27.38%), and Web of Knowledge (924/3298, 28.02%), as well as snowball sampling (95/3298, 2.88%) and manual searching (255/3298, 7.73%). The selection process consisted of 3 key stages: title-abstract screening, disagreement resolution, and full-text screening. The initial steps included removing 2066 (62.64%) of the 3298 duplicate records, followed by the exclusion of 1.48% (49/3298) of records because of publication issues, such as mismatched publication year or language. This resulted in 35.87% (1183/3298) of the records eligible for screening. During the title-abstract screening, 78.19% (925/1183) of the records were excluded for not meeting the inclusion criteria, leaving 258 (21.81%) papers for full-text screening. In this final stage, a further 46.9% (121/258) of the records were excluded, culminating in 137 (53.1%) studies included in the review (refer to Multimedia Appendix 3 for more information).

The triangulated approach to identifying, selecting, and retrieving studies ensured a robust and comprehensive review, including only the most relevant and high-quality studies. These systematically categorized studies provide a solid foundation for both quantitative and qualitative analyses, addressing key research questions on patient similarities across definitions, methodologies, data sources, and cancer types. The analysis of the resulting 137 studies provides critical insights into patterns of patient similarity, creating a comprehensive understanding of the current state of cancer research and care.

### Multidimensional Analysis of Similarity in Patients With Cancer

#### Overview

**Figure 2:**
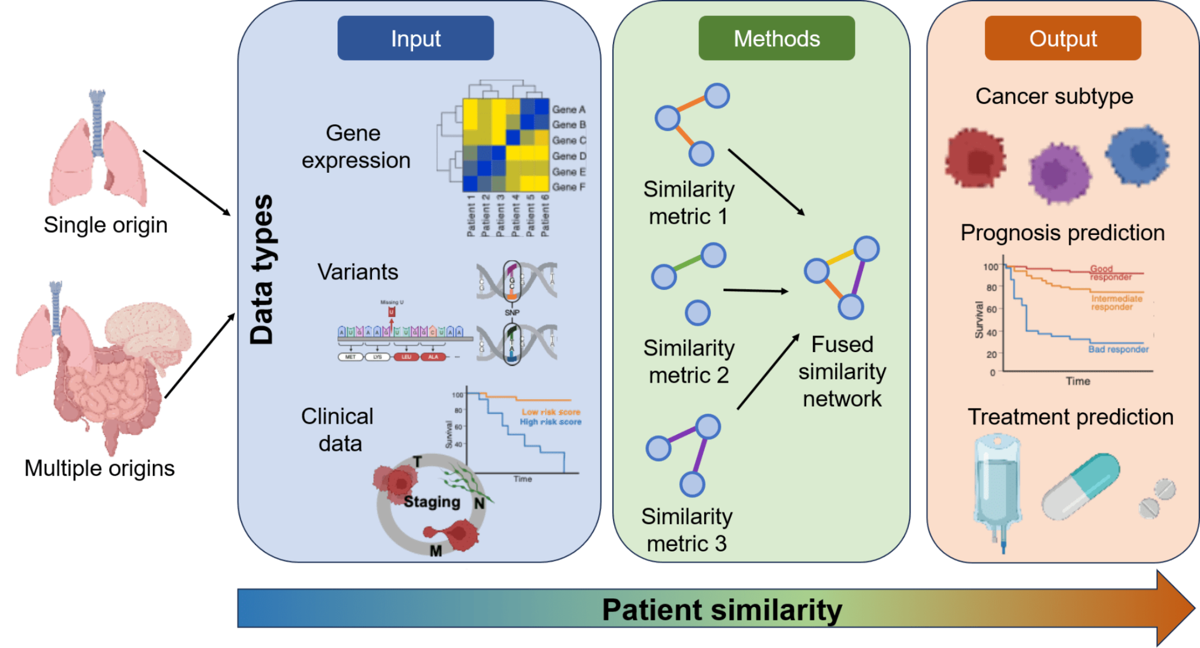
Framework for identifying similarities between patients with cancer. Input data that can be used for patient similarity can come from a single source or organ or multiple organs (origins), depending on the use case. The different similarity metrics used can be applied to various types of data, such as gene expression, variants, or clinical data (survival and tumor staging). Note that not all similarity metrics can be applied to all data types. After defining similarity metrics, they are fused into a similarity network that can be used to categorize cancer subtypes, to predict the prognosis of patients with cancer, or to determine the most appropriate next treatment.

The literature overview highlights that the identification of similarities in patients with cancer requires a multidimensional approach rather than a simple linear one. This analysis integrates 4 key dimensions: defined knowledge, analytical methods, cancer types, and available datasets (Figure 2). These dimensions form the basis of a framework for a comprehensive understanding of similarities in patients with cancer and their metrics. Each dimension provides a unique contribution: defined knowledge establishes the theoretical foundation, analytical methods offer tools for processing and interpreting data, cancer types supply biological specificity, and available datasets define the scope and granularity of the analysis (Figure 2). Together, these dimensions enable a comprehensive and systematic exploration of patient similarities, reflecting the complexity and variability of cancer research. We assert that only such a multilayered perspective can effectively define and measure *similarities in patients with cancer* in this intricate field (Figure 2).

#### Approaches and Methods for Identifying Similarities in Patients With Cancer

##### Overview

Building on this framework, a structured categorization of analytical methods was conducted to identify the primary approaches used in cancer similarity studies. These methods were grouped into 3 categories: machine and deep learning approaches, network- and graph-based approaches, and statistical and advanced mathematical techniques. Of the 137 publications, machine and deep learning approaches were the most frequently represented, appearing in 76 (55.4%) studies. This was followed by network- and graph-based approaches in 72 (52.5%) publications and statistical and advanced mathematical techniques in 60 (43.7%) publications.

##### Machine and Deep Learning Approaches

Machine and deep learning approaches are the most prominent methods for analyzing patient similarity. Their scalability enables them to process extensive datasets, which is crucial given the complexity and volume of data in cancer research. These approaches excel in predictive and classification tasks, such as predicting patient outcomes, classifying cancer types, and identifying cancer subtypes.

Table 1 shows the distribution of methods identified in the reviewed publications, grouped into 3 method types. The most common machine and deep learning approach found was spectral clustering, appearing in 8% (11/137) of the studies [24-32]. Hierarchical clustering was also a commonly used method (9/137, 6.6%) [33-39]. In addition, consensus clustering was observed in 5.8% (8/137) of the studies (Table 1) [39-45,160]. Beyond the methods summarized in Table 1, the classical application of support vector machines was used in various tasks, including biomarker identification [46-49], drug response prediction [48], and patient similarity measurement [46,47,50]. Federated learning was also highlighted for enabling multiple institutions to collaboratively develop machine learning models while maintaining data privacy [161]. Other machine learning approaches included recursive feature elimination based on the support vector machine and DeepLIFT (deep learning important features) [51]. Lee et al [52] proposed a framework for federated learning for patient similarity learning.

**Table 1:** Quantitative analysis of papers using specific methods in each method category (N=137).

Method category and methods		Papers, n (%)
**Machine and deep learning approaches**		
	Spectral clustering	11 (8)
	Hierarchical clustering	9 (6.6)
	Consensus clustering	8 (5.8)
	K-means clustering	6 (4.4)
	Graph convolutional network	5 (3.6)
**Network- and graph-based approaches**		
	Patient similarity network	30 (21.9)
	Similarity network fusion	19 (13.9)
	Protein-protein interaction network	11 (8)
	Weighted gene coexpression network analysis	3 (2.2)
	Affinity matrix	2 (1.4)
**Statistical and advanced mathematical techniques**		
	Nonnegative matrix factorization	4 (2.9)
	Bayesian predictive models	2 (1.4)
	Sign algorithm	2 (1.4)
	Semantic similarity	2 (1.4)

##### Network- and Graph-Based Approaches

Network- and graph-based approaches focused on modeling relationships and interactions within data, such as patient similarities derived from shared genetic markers or biological pathways, leverage various network and graph constructs. These include patient similarity networks (PSNs) and protein-protein interaction (PPI) networks, supported by analysis techniques such as similarity network fusion (SNF) and graph convolutional networks (GCNs). Their primary strength lies in their capacity to model complex systems, making them highly valuable for identifying patient subgroups and enabling classification and treatment stratification.

**Figure 3:**
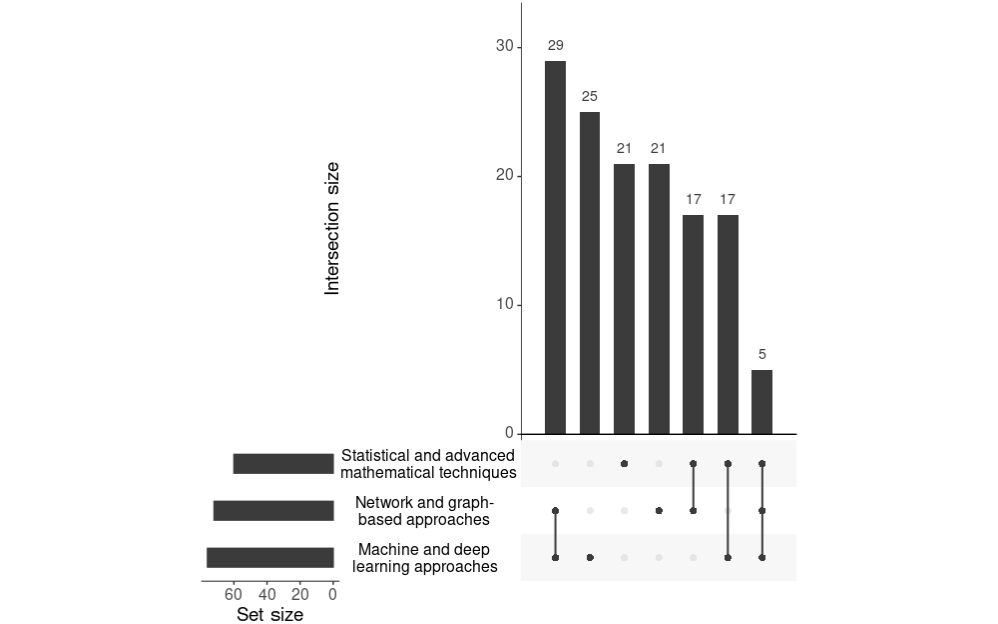
Methodological intersections in patient similarity analyses. The UpSet plot shows how frequently different categories of computational approaches—statistical and mathematical techniques, network- and graph-based approaches, and machine and deep learning—are used alone or in combination in studies on similarity in patients with cancer. Horizontal bars indicate the total number of studies applying each method type; vertical bars represent the size of intersections between method categories.

A detailed analysis of this category showed that PSNs were used in 21.9% (30/137) of the publications, making them the most frequently used method within this category (Figure 3) [28,29,44,49,53-59]. In PSNs, as described by Pai and Bader [60], nodes represent individual patients, while edges denote pairwise similarity based on selected data features, such as clinical or genomic data. Calculating edge weights involves using specific patient similarity measures. The Pearson correlation coefficient is a robust measure for this purpose, as it remains effective even when clinical datasets contain missing values—a common issue because of variations in DNA sequencing panels. Separate networks can be constructed for each feature, providing distinct *views* of patient similarity. These feature-specific networks are instrumental in identifying patient subgroups by detecting clusters of closely related individuals and can also be used to develop predictive models. A significant advantage of PSNs over many machine learning approaches is their high interpretability. Moreover, PSNs can be integrated with other methods, such as SNF, to enhance their applicability and utility [60].

The second most frequently used method in our selected publications is SNF (19/137, 13.9%) [28,29,49,53-57,59,61,62]. The basic principles for SNF were first described by Wang et al [15]. SNF is a method for integrating different data types, such as messenger RNA expression, DNA methylation, or copy number variations, for the same set of samples. It first constructs a PSN for each data type, where edge weights are represented by a similarity matrix W. The similarity between patients i and j is stored in W(i, j) [15]. Subsequently, SNF iteratively fuses these networks using a nonlinear method based on message-passing theory, resulting in a single network that captures the relationships across all data types. The fused network is then used for clustering to identify subtypes, capturing both similarities and differences between the samples. SNF excels at combining strong similarities from individual networks while reducing noise and retaining weak similarities consistent across all data types [15]. Several extensions to the SNF approach have been proposed. Wang et al [54] and Li et al [61] trained a GCN on the similarity matrix derived from SNF. This combined approach was used to predict the survival time of patients with cancer [54]. Zhang et al [53] combined SNF with dense GCNs (DenseGCNs) for liver cancer diagnosis. DenseGCNs improve information flow within the network by densely connecting different layers, which can alleviate the vanishing gradient problem [53].

Affinity network fusion (ANF) is another modification of SNF. In ANF, similarity networks are calculated for each data type using a distance metric, followed by a local Gaussian kernel and a k-nearest neighbor graph with subsequent normalization and pruning of weak edges. Rather than iteratively fusing affinity networks, ANF performs 3 random-walk iterations by definition, and thus, it saves computation time [162]. ANF was successfully used to cluster patients with cancer [24,162].

PPI networks are another frequently used method in our selected studies, with 8% (11/137) of instances [63-68]. In a PPI network, PPI data are used to map interaction networks based on physical or functional connections [163]. In these networks, proteins or protein-coding genes are represented as individual nodes, and an edge is drawn between interacting proteins to indicate the presence of a physical interaction [65]. Analyzing interactions of proteins encoded by cancer genes aids in identifying novel candidate genes and improving prioritization methods for these genes [164].

GCN methods for semisupervised learning on graph-structured data, such as networks, appeared in 3.6% (5/137) of the selected publications [53,54,61,69,70]. As proposed by Kipf and Welling [165], GCNs extend convolutional neural networks to graph-structured data, capturing and leveraging relationships between objects within the graph.

Weighted gene coexpression network analysis (WGCNA) is another network-based method frequently applied to analyze how genes jointly contribute to complex human diseases. WGCNA constructs a gene coexpression network represented by an adjacency matrix, where elements denote the similarity of coexpression between a pair of genes. Hierarchical clustering is then applied to identify closely linked genes, known as gene modules. Hub genes—those with extensive interactions—are identified within these modules. Identified modules can also be associated with disease phenotypes by correlating the module eigengene (the first principal component of the module) with the trait [71,166]. For example, Huang et al [71] used WGCNA to identify gene modules, which were subsequently used to identify gene signatures. Similarly, Zhang and Sun [72] applied WGCNA to identify hub genes within modules.

##### Statistical and Advanced Mathematical Techniques

Statistical and advanced mathematical techniques involved mathematical models and statistical tests to provide theoretical foundations for other methodologies (Figure 3). This category includes approaches such as probability theory, regression analysis, and hypothesis testing. Although these methods were used less frequently compared to others, their fundamental role in validating findings and ensuring rigorous data analysis cannot be overstated. These techniques often complement other approaches by providing a robust statistical framework, supporting model accuracy, and refining the overall analysis.

Publications were categorized under statistical or advanced mathematical techniques if these methods played a significant role in shaping the methodology used within the study. Methods that were not already assigned to another category were assigned to this category. For example, while machine learning or network-based methods are based on mathematical principles, they were then assigned to their corresponding methods.

A frequently used approach is principal component analysis [26,63,73-75,167], a technique designed to reduce the dimensionality of a dataset while preserving its most significant information. The process starts with the computation of a covariance matrix, followed by calculating its eigenvalues and eigenvectors. The eigenvectors represent the principal components, while the eigenvalues indicate the proportion of variance explained by each component. The number of principal components equals the number of original variables, but typically, a subset is selected that represents the largest proportion of the variance in the dataset. This is done by selecting the eigenvectors with the highest eigenvalues. The data are then projected onto the selected principal components, achieving dimensionality reduction [168]. In the reviewed publications, principal component analysis was applied to reduce noise [75], extract the most informative molecular signatures [63], or reduce computational effort for subsequent methods [167].

A different approach for dimension reduction is nonnegative matrix factorization (NMF). Both NMF and its extension, seminonnegative matrix trifactorization (NMTF), are techniques for analyzing and understanding nonnegative data matrices. They decompose the data matrix into a set of nonnegative factors, revealing underlying patterns and structures. NMF decomposes the matrix into 2 nonnegative factor matrices, and NMTF into 3 matrices. The process involves optimizing the factor matrices to minimize a cost function. However, this optimization problem cannot be solved analytically and must be approached using numerical methods, such as iterative algorithms [169,170]. In the previewed publications, NMF [76-78] or NMTF [41,79] were used to enhance clustering approaches.

Another method used to reduce dimensionality while preserving significant information is the minimum redundancy and maximum relevance feature selection algorithm [54]. The technique aims to identify a subset of features that are both highly relevant to the problem and minimally redundant. This is achieved by simultaneously maximizing the relevance and minimizing the redundancy of the selected features [171].

Similarity identification in gene expression offers an approach to uncover patient similarities [80,81] by leveraging gene expression data within the context of their biological pathways. This approach constructs gene expression matrices specific to each pathway and calculates the transcriptional similarity coefficient, a metric ranging from −1 to 1 that quantifies the similarity in pathway activity between 2 patient samples [81]. Similarity identification in gene expression has proven effective in predicting overall survival rates in patients with breast cancer [81] and in identifying drug response biomarkers specific to the HER2+ subtype [80].

Semantic similarity is another approach used to determine similarity [82-84,172] by evaluating the resemblance between texts or text excerpts. For example, it has been applied to calculate similarity between diseases using Medical Subject Headings descriptions [84] or between clinical documents using semantic vectors [172]. Gene ontology terms have also been frequently used to determine the semantic similarity between patients [83] or genetic features [82].

#### Mapping the Overlaps of Methodological Categories

The multifaceted nature of cancer research necessitates the integration of various methodological approaches, resulting in significant overlaps between method categories (Figure 3). These overlaps reflect the interdisciplinary strategies used to analyze patient similarities and address the complexity of cancer data. This section examines these overlaps in detail, emphasizing their implications for advancing cancer research.

Figure 3 illustrates that machine and deep learning approaches, along with network- and graph-based methods, frequently co-occur with methodological categories, observed in 21.2% (29/137) of the studies. This combination is particularly common in research on cancer subtype identification, where similarity networks are typically constructed as a foundation for subsequent clustering algorithms. The integration of these methods underscores their complementary strengths: machine learning excels in pattern recognition and prediction, while network-based methods effectively model data relationships, thereby enhancing predictive power and facilitating the discovery of complex biological interactions. In 12.4% (17/137) of the studies, network- and graph-based approaches were combined with statistical and advanced mathematical techniques, illustrating the frequent use of statistical tools to validate network models. This combination enhances the reliability of insights derived from relational data analysis, particularly in understanding cancer-related biological interactions. The intersection of machine learning and statistical techniques was also identified in 12.4% (17/137) of the papers, underscoring the necessity of statistical validation in machine learning models. Statistical techniques enhance the interpretability and robustness of machine learning outputs, ensuring reliable and reproducible findings in cancer research. While all 3 methodological categories overlapped in only 3.6% (5/137) of the studies, this integration provided a comprehensive framework for cancer data analysis, enabling precise survival analyses and pathway identification. These findings underscore the value of combining methods to tackle the complexity of cancer datasets.

### Analysis of Data Types

As specified in one of the research questions of this scoping review, data sources play an important role in defining patient similarity in cancer research. These sources can be seen as a distinct dimension of patient similarity or as part of an interconnected framework encompassing 4 dimensions that collectively define this concept. Given the complexity of cancer and its numerous influencing factors, a wide variety of data sources is used. To streamline this diversity, we categorized the data into 6 broad groups: genetic and genomic data, clinical data, transcriptomic data, proteomic and metabolomic data, epigenetic data, and 1 functional category—pathway and network data. Table 2 presents the classification of data types with examples, while Multimedia Appendix 4 provides a visualization of their distribution.

**Table 2:** Categories of detected data and representative examples.

Data category	Examples of corresponding data represented in the paper
Genetic and genomic data	CNV^a^, SNV^b^, somatic mutations, variant data, gene ontology and molecular functional profiles, and gene interactions
Epigenetic data	DNA methylation and hypermethylation
Transcriptomic data	Transcriptome (mRNA^c^ expression, exon expression, microRNA arm-switching, lncRNA^d^, RNA microarray, RNA sequencing, scRNA-Seq^e^, transcription factors, and gene signatures)
Proteomic and metabolomic data	Proteomics, metabolomics, and mass spectrometry data
Clinical data	Primary site, tumor grade, maximum tumor size, number of lesions, locations of lesions, and MSI^f^, MMR^g^ status, lymph node status and information, drug substructure fingerprints, drug resistance, drug-exposure gene expression data, chemical compound activity data, and histology
Pathway and network data	Pathway features and aberration profiles (mRNA pathways, pathway activities, and PPI^h^)

^a^CNV: copy number variation.

^b^SNV: single nucleotide variation.

^c^mRNA: messenger RNA.

^d^lncRNA: long noncoding RNA.

^e^scRNA-Seq: single-cell RNA sequencing.

^f^MSI: microsatellite instability.

^g^MMR: mismatch repair.

^h^PPI: protein-protein interaction.

**Figure 4:**
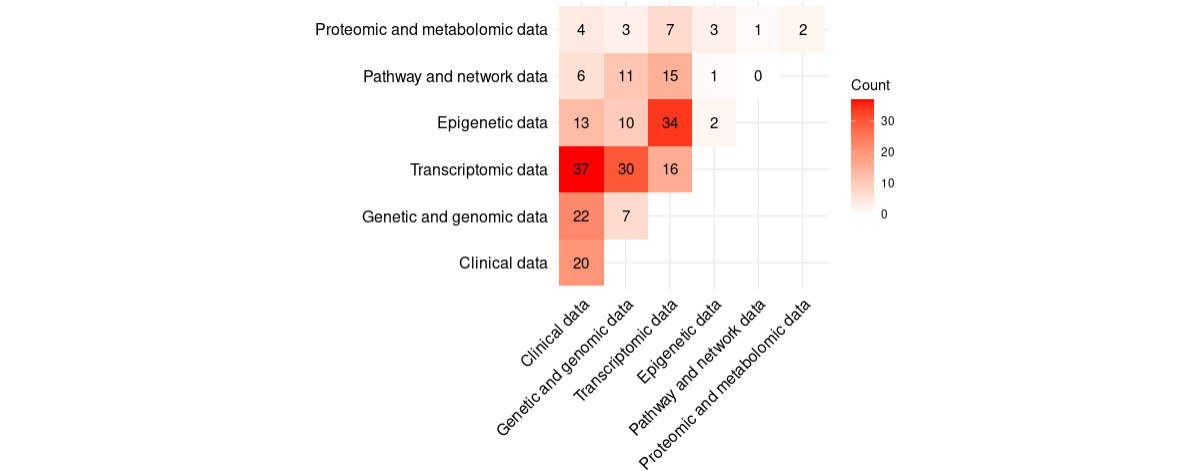
Co-occurrence of data types used in patient similarity studies. The matrix displays pairwise combinations of data categories, with cell values indicating the number of studies that used both types. Darker red shading reflects higher co-occurrence frequencies, highlighting common integrative data combinations such as transcriptomic with clinical or genomic data.

It is important to note that data types in cancer research are rarely used in isolation; instead, they are often combined to achieve a more comprehensive and meaningful representation of patient similarity. The choice of data types to be combined depends largely on the research objectives and the methodologies applied. Therefore, we analyzed the data from different perspectives, considering not only their absolute abundances but also the frequencies of specific combinations (Figure 4). The most frequently used data type is transcriptomic data, accounting for 67.1% (92/137) of the studies. This category includes expression data for microRNA, messenger RNA, and exons [85,173,174], as well as gene signatures and microarray data [73,86], reflecting its critical relevance in cancer research, particularly in biomarker discovery and subtype identification [53-55,73,86]. These data are most often used in combination with other types, particularly clinical data (37/137, 27%) [53,174] and epigenetic data (34/137, 24.8%) [54,55]. The frequent overlap between transcriptomic and clinical data reflects the relevance of gene expression patterns in relation to patient-specific clinical features, providing molecular insights that complement phenotypic observations. Similarly, transcriptomic data are often combined with genetic and genomic data (30/137, 21.9%; Figure 4), highlighting the influence of genetic alterations, such as mutations and copy number variations, on downstream gene expression. This integration enhances the understanding of molecular mechanisms driving cancer progression and underscores the importance of combining these data types for comprehensive similarity analyses. Clinical data, the second most common category, is highly heterogeneous, limiting the interpretive significance of its frequency alone. In addition to image data, this category includes data on the activity of chemical compounds [56], tumor size, and blood counts [5]. The methodological approaches described in the associated papers are often explicitly tailored to a data group in this category. While clinical data are often used in combination with transcriptomic data (Figure 4), they also appear as the most frequent stand-alone category (20/137, 14.5%). The versatility of clinical data lies in its ability to provide a contextual framework for other molecular data types, despite the analytical challenges posed by heterogeneity [56,87]. Another important category is genetic and genomic data. This includes studies investigating copy number variations [57], gene interactions [88], and molecular profiles [49]. With a frequency of 35% (48/137), this data group is the third most common and often appears in combination with clinical data (20/137, 14.6%) [44,49] and transcriptomic data (30/137, 21.9%) [28,29,59]. This may be because genetic alterations, such as copy number variations and mutations, can directly influence gene expression. For example, increased gene copy number (amplification) can lead to overexpression of an oncogene, which promotes tumor growth [63]. Therefore, when both types of data are considered together, the chance of obtaining a more detailed picture of mutational influences for similarity analysis is significantly higher. Epigenetic data, which account for approximately 27% (37/137) of the studies, are mainly used in combination with transcriptomic data (34/137, 24.8%; Figure 4) [54] and are rarely used as a stand-alone data source (2/137, 1.4%) [64]. DNA methylation profiles, which are part of this category, are key epigenetic features that influence cellular phenotypes and patient similarity. However, the impact of methylation often becomes more apparent when integrated with other types of molecular data, highlighting the complex interplay between epigenetic and genetic factors [65]. Proteomic and metabolomic data (10/137, 7.3%) and pathway and network data (18/137, 13.1%) make up the smallest proportion of the types of data sources used. These include proteomics [66], metabolomics [67], and mass spectrometry data [68], as well as PPI data [163] and pathway aberration profiles [164]. Notably, pathway and network data are only used in combination with other data types (Figure 4). In other words, they serve in a supporting or augmenting capacity but are not used independently to determine similarity. However, very innovative approaches in this data category have led to progress, especially in the areas of simulation and prediction of survival [163,164]. Quantitatively, transcriptomic data, that is, expression data, were analyzed particularly frequently, while genetic and genomic data were studied significantly less often. This may be because of the higher clinical relevance of expression data [61]. This prioritization may indicate that transcriptomic data are considered a more direct indicator of disease status and treatment response. In addition, the methodological approaches for the analysis of expression data are often more mature and better established, which supports their frequent use. In contrast, genetic and genomic data provide deeper insights into the underlying mechanisms of carcinogenesis but are often only fully interpreted in combination with other data types. The heterogeneity of clinical data underscores their versatility but also makes them difficult to analyze in a consistent manner. The variety of data sources used reflects the complexity of cancer research, which requires a multidisciplinary approach.

### Interdependence of Data Types and Methods

A review of the types of data commonly used in similarity studies involving patients with cancer emphasizes the intrinsic link between data types and methodological approaches. The classification of methodological aspects of patient similarity is inherently dependent on the type of data used. The type of data directly affects how similarity can be calculated and interpreted. This relationship can be understood by examining the underlying principles of methods, their fields of application, and the specific types of data they use. Consequently, it is important to analyze the interaction between methodological categories and the data used in the context of patient similarity, highlighting the strong correlation between data parameters and methodological parameters. After examining this interrelation, we conclude that machine and deep learning methods are most prominently applied to transcriptomic, clinical, and genomic data. This analysis also shows that network- and graph-based approaches are applied to the same data as machine learning models, while slightly favoring pathway and interaction data.

### Frequently Researched Cancer Types

To analyze the cancer types that have been frequently studied in relation to patient similarity, we evaluated the number of papers using datasets from specific cancer types. Many papers, especially those focused on methodological innovation, use large and diverse cancer datasets that include >5 major cancer types [24,33,89,90]. However, several papers focus extensively on 1 or 2 major cancer types, or even on 1 or 2 cancer subtypes [40,91-93]. These studies often use methods specifically designed for these entities to obtain specific information [40,91-93].

All these publications share 1 commonality: several main cancer types and subtypes appear frequently, either as part of a larger dataset or on their own. We categorized the cancer types by primary tumor site using OncoTree [159]. We identified 5 commonly reported primary tumor sites: kidney, bowel, central nervous system, that is, brain, lung, and breast, as outlined in Table 3.

**Table 3:** Most frequently investigated primary tumor sites and entities in the patient similarity analysis (N=137).

Primary tumor sites and entities		Papers, n (%)
**Primary tumor sites**		
	Breast	72 (52.5)
	Lung	38 (27.7)
	Central nervous system or brain	32 (23.3)
	Bowel	31 (22.6)
	Kidney	29 (21.2)
	Ovary or fallopian tube	19 (13.9)
	Hematologic cancer (lymphoid and myeloid)	17 (12.4)
	Liver	15 (10.9)
	Prostate	13 (9.5)
	Head and neck	12 (8.7)
	Esophagus or stomach	12 (8.7)
	Cervix	9 (6.6)
	Bladder	8 (5.8)
	Skin	7 (5.1)
	Uterus	7 (5.1)
	Pancreas	6 (4.4)
	Sarcoma (soft tissue or bone cancer)	6 (4.4)
	Thyroid	5 (3.6)
	Bladder or urinary tract	5 (3.6)
**Entities**		
	Breast invasive carcinoma (BRCA)	49 (35.8)
	Glioblastoma multiforme (GBM)	28 (20.4)
	Kidney Renal Clear Cell Carcinoma (KIRC/CCRCC)	24 (17.5)
	Lung squamous cell carcinoma (LUSC)	22 (16)
	Colon adenocarcinoma (COAD)	19 (13.9)
	Ovarian serous cystadenocarcinoma (OV)	15 (10.9)
	Lung adenocarcinoma (LUAD)	12 (8.7)
	Head and neck squamous cell carcinoma (HNSC)	11 (8)
	Liver hepatocellular carcinoma (HCC)	9 (6.6)
	Bladder urothelial carcinoma (BLCA)	9 (6.6)
	Acute hematologic cancer (lymphoid and myeloid)	8 (5.8)
	Leukemia (LAML/AML)	8 (5.8)
	Prostate adenocarcinoma (PRAD)	8 (5.8)
	Cervical squamous cell carcinoma (CESC)	7 (5.1)
	Skin cutaneous melanoma (SKCM)	6 (4.4)
	Kidney renal papillary cell carcinoma (KIRP)	5 (3.6)
	Stomach adenocarcinoma (STAD)	5 (3.6)
	Sarcoma (SARC)	5 (3.6)

With a conspicuously large quantity of 52.5% (72/137) mentions in different publications [28,66,94,175], breast cancer is the most studied cancer regarding patient similarity. This is logically consistent with the fact that breast cancer is also widely studied in different cancer research fields because of its high prevalence in the population [176] and the abundance of well-categorized data available [177]. The gap between breast cancer and the second most studied type, lung cancer (38/137, 27.7%) [30,41,95,96], underscores the prominence of breast cancer in cancer research. This is further confirmed when looking at specific cancer subtypes, where breast invasive carcinoma (BRCA) is generally the most researched subtype (49/137, 35.8%), although in most cases, the specific BRCA type was not mentioned in the publications [24,25,97,98]. Interestingly, the second and third most studied specific cancer types are glioblastoma multiforme (GBM) and renal clear cell carcinoma (clear cell renal cell carcinoma). GBM was studied in 20.4% (28/137) of the publications [26,42,57,99] and represents the majority of brain cancers studied. The same can be observed with kidney renal clear cell carcinoma or clear cell renal cell carcinoma: 83% (24/29) of the total mentions of bowel cancer refer to this cancer [25,34,41,100].

The specific lung cancers studied are divided into 2 main types—lung squamous cell carcinoma (LUSC) and lung adenocarcinoma—with LUSC being more common, with 16% (22/137) mentions, than lung adenocarcinoma, with only 8.7% (12/137) mentions [30,41,96]. In bowel cancer, colorectal adenocarcinoma was the most commonly reported specific type with 13.9% (19/137) mentions [32,34,41,90]. It is also striking that ovarian serous cystadenocarcinoma and acute hematologic cancers (eg, lymphoid and myeloid cancers), which are less common than some other cancers but have been the focus of significant research, are also listed (15/137, 10.9% and 8/137, 5.8%, respectively). However, in most cases, they were only part of a larger dataset that included >5 different cancers (11 of 15 and 6 of 8, respectively), which relativizes the true importance of the findings in this context.

Overall, the most commonly studied cancer types regarding patient similarity are breast cancer (especially BRCA), followed by central nervous system or brain cancer (especially GBM) and lung cancer (especially LUSC). Including kidney and bowel cancer, the top 5 primary affected tumor sites account for >50% of the total number of mentions of cancer types in the studies.

### Overview of Similarity Metrics

We identified several similarity metrics frequently used in cancer research to compare patient data. Patient similarity is context dependent, influenced by specific methods, goals, and expert perspectives [101,102]. Common approaches represent patients as vectors in feature spaces, with similarity expressed as a distance metric [5,12,18,178]. The multidimensional feature spaces are often divided into different data categories that are considered differently in the similarity calculation, such as numerical data, binary data, and classification data or structured data (eg, gender and age) and unstructured data (eg, diagnostic texts) [12,102]. Using this data concept, patient similarity can be understood as the distance between 2 vectors in the feature space, which can be intuitively described as the edge weight on the edge between 2 nodes in a graph, as is the case in methods such as the PSN [60,88,101,179]. Depending on the approach, it is possible to examine all features together or to evaluate multiple features individually and ultimately merge them [5]. This representation can be used to define distances between the vectors of individual patients using mathematical methods.

There are several distance or similarity measures that are used to compute similarities. The most common metrics are the Euclidean distance [27,60,87,88,101,179], cosine similarity [18,60,87,88,101,103,178,179], Jaccard similarity [87,88,101,178], and Pearson correlation [60,85,88,101,103,104,179]. Cosine similarity calculates the cosine of the angle between 2 patient vectors in feature space. The calculation can be simplified using the dot product described by Brown [87]. The result is in a range between −1 and 1, where 1 is the maximum similarity [87,178]. The Jaccard similarity measures the similarity of 2 sets compared to each other. It describes the coefficient of the size of the intersection and the size of the union of 2 sets. The result ranges from 0 to 1, where 1 represents the optimal similarity between 2 patients. This concept can be used to compare symptom phenotypes of patients [27,173]. The Pearson correlation (or Pearson correlation coefficient) is a measure of the linear relationship between 2 variables. It ranges from −1 to 1, where 1 indicates a perfect positive correlation, −1 indicates a perfect negative correlation, and 0 indicates no correlation [104,174]. This similarity metric is particularly robust to missing data within the patient vectors, making it an ideal tool for analyzing incomplete datasets [60,179]. Other similarity measures, such as Hamming, City Block, Minkowski, and Chebyshev distances, are also mentioned [88,101]. Overall, the specific measures are usually the basis for individually defined distances, which are also adapted to the data sources used. For example, when using gene and microRNA expression levels, the distance between 2 samples can be calculated using the Euclidean distance in combination with other metrics [18,27,87,103]. However, depending on the type of data available, new distance measures may need to be used. For example, Virgolin et al [86] describes distance measures that focus on the physical evaluation of image data. They define different similarities based on organ deformation, organ overlap, organ shape, and organ constellation. Even these approaches often use known coefficients and metrics, such as the Hausdorff distance, as a starting point [86].

When creating a similarity score, it is very important to consider the characteristics of the data and to optimize the metric according to the underlying goals. For example, if the goal is to identify driver genes and compare patients for similar mutations, the metric must be able to represent the similarity of mutated genes in patients [105]. In this specific case studied by Zhang et al [73], the similarity of the Gaussian interaction profile kernel was used to score functional similarity. When numerical data alone are not available, as is often the case with clinical data, ways must be found to incorporate both continuous and binary features. This can be done by defining a similarity measure for each data category [5] and then scoring them individually, or by using metrics such as the Gower similarity coefficient for the categories and merging them using the geometric mean calculation [88]. However, the more diverse the data types, the more difficult it becomes to make an all-encompassing statement limited to 1 value, which is why the development of methodological approaches for determining similarity between patients plays such an important role. Overall, defining patient similarity in cancer research involves understanding the interplay between different clinical, biological, and treatment data types. The complexity and heterogeneity of cancer underscore the need for a multifaceted approach, ensuring that similarity metrics adequately reflect the nuanced relationships between patients.

### Research Objectives of Similarity Identification Between Patients With Cancer

**Figure 5:**
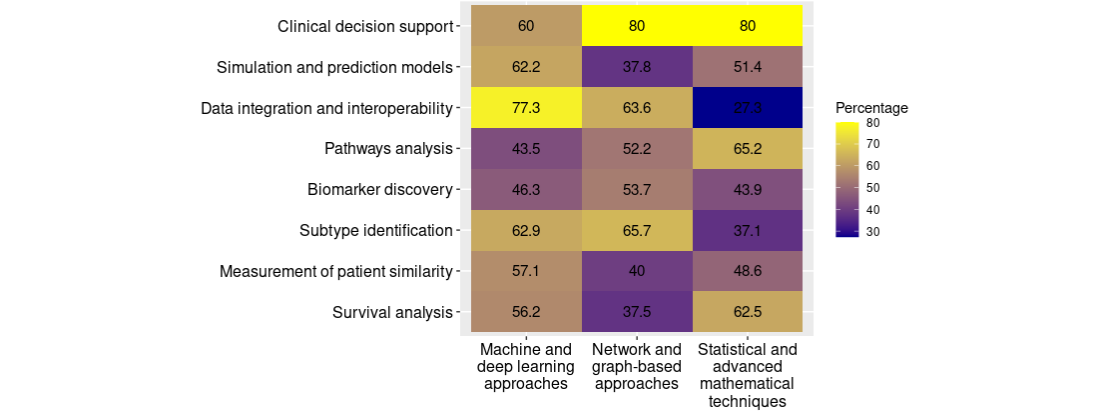
The use of computational method categories across different research aims in cancer-related patient similarity studies. The heat map displays the percentage of studies applying machine and deep learning, network-based, or statistical techniques to various biomedical objectives, including clinical decision support, survival analysis, and subtype identification. Higher percentages (yellow) indicate stronger associations between method types and specific research purposes.

Our ongoing analysis has identified several key objectives frequently applied to define similarity in patients with cancer, as shown in Multimedia Appendix 5. Most of our publications addressed the identification of cancer subtypes, with 51.1% (70/137) of the publications focusing on this objective. As cancer is a highly heterogeneous disease, there are many subgroups that may respond differently to various therapies, allowing different treatment methods to be chosen [24,180]. Patients within a subtype are, by definition, similar and can therefore benefit from the treatment outcomes of other patients. To identify these subtypes, machine and deep learning methods (44/70, 63%) as well as network- and graph-based approaches (46/70, 66%) were used predominantly, as shown in Figure 5 [24,25,27,31].

**Figure 6:**
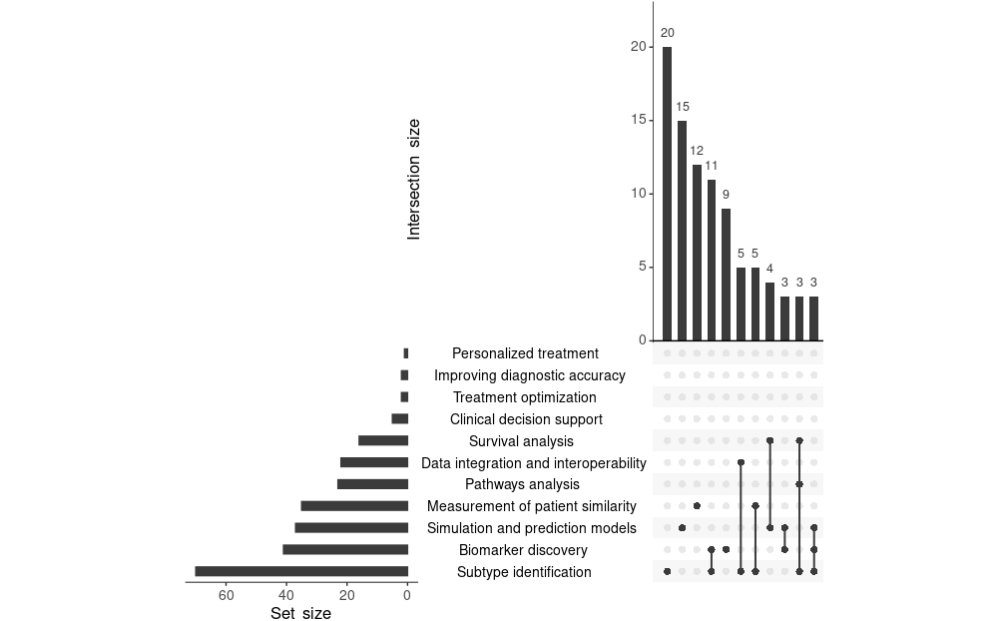
The intersections among research objectives used to analyze similarities. The UpSet plot visualizes how frequently different analytical goals, such as subtype identification, biomarker discovery, or clinical decision support, are addressed alone or in combination across studies. Vertical bars show the number of studies sharing the respective combinations of objectives, while horizontal bars indicate the total number of studies associated with each individual objective.

The second most common aim of the analyzed publications was biomarker discovery [46,49,64,65,72,97,106], which was the focus of 29.9% (41/137) of the publications. According to the National Cancer Institute, biomarkers are biological molecules that serve as indicators of normal or abnormal processes or diseases. They play an important role in the diagnosis and treatment of patients with cancer [181], and the discovery of new biomarkers is crucial for improving the success rate of therapies [68]. The transition between the identification of subtypes and biomarkers is fluid, as subtypes are often defined by specific biomarkers. This explains the significant overlap detected between these 2 patterns of similarity identification (Figure 6). A notable overlap was observed between subtype identification and the measurement of patient similarity, as subtypes often reflect patient-specific features that define similarity patterns. This connection highlights the interdependence of these 2 concepts in patient stratification efforts. The distribution of machine and deep learning, as well as network- and graph-based approaches, shows a relatively balanced representation. Similarly, a notable overlap was identified between subtype identification and data integration, which is logical, as integrating diverse data types often aids in identifying complex subtype-specific characteristics. Moreover, overlaps between subtype identification and simulation or prediction models were prominent, reflecting the reliance of predictive analyses on subtype-specific features (Figure 6). Predictive models frequently used these features to predict survival outcomes or drug responses. In addition, overlaps were observed between simulation and prediction models and survival analysis, indicating that survival outcomes are often subtype dependent (Figure 6). Simulations and prediction models were also frequent, with 27% (37/137) of the publications using them. In particular, machine and deep learning approaches [48,49,54,169] were used (23/37, 62%; Figure 5). Different approaches to predictive models were used. For example, in some cases, survival time was predicted [38,55,69,107]. Other prediction models included drug response prediction [48,62,80,108] and diagnosis prediction [53]. Because predictive analyses frequently rely on subtype-specific features, a notable overlap was identified between subtype identification and simulation or prediction models (Figure 6). Measurement of patient similarity emerged as another crucial area of research, with 25.5% (35/137) of the publications focusing on this aspect. A clear overlap with subtype identification was detected, as subtypes are often defined by shared similarity metrics across patient groups. Simulations and prediction models were followed at some distance by pathway analysis, but with considerably lower frequency, with 16.8% (23/137) of the publications. Many of these publications focused on the identification of cancer-related pathways [74,92,109]. Data integration and interoperability were addressed in 16% (22/137) of the publications, while clinical decision support was the focus in 3.6% (5/137) of the publications. Furthermore, overlaps were observed between pathway analysis, subtype identification, and survival analysis, emphasizing the role of pathway-specific mechanisms in subgroup survival outcomes. As shown in Figure 6, overlaps were also observed between pathway analysis and both biomarker discovery and subtype identification. This indicates the importance of pathway analysis in identifying biomarkers and understanding mechanisms within subgroups.

## Discussion

### Overview

This scoping review offers a comprehensive synthesis of how patient similarity is defined, operationalized, and applied in current cancer research. The analysis identified 4 central dimensions that collectively shape the conceptualization and implementation of the patient similarity model: research objectives, analytical methods, cancer type representation, and data availability. Rather than representing a fixed or uniform metric, this study highlights that patient similarity is a dynamic construct shaped by the interaction of multiple elements and influenced by methodological choices and clinical intent.

### Principal Findings

A key finding of this review is the multifaceted nature of patient similarity in oncology. The analysis of this multidimensional modeling approach revealed that similarity is applied across a range of research objectives, including subtype identification, biomarker discovery, prediction modeling, survival analysis, and pathway analysis. Among these, subtype identification and biomarker discovery were the most prevalent, appearing in more than half of the reviewed studies. This emphasis highlights the central role of patient similarity in optimizing stratification and informing therapeutic interventions, an imperative that aligns with the heterogeneity of cancer and the need for individualized treatment strategies. This focus can be explained by the fact that identifying molecular subtypes and actionable biomarkers remains a foundational step in precision oncology. It allows for the classification of patients into clinically relevant categories that may respond differently to therapies, thus improving outcomes and resource allocation [24,25,46,49,64].

Beyond identifying research objectives, this review provides insight into how patient similarity is measured and conceptualized. It was observed that the selection of similarity metrics varied significantly across studies, depending largely on specific research aims and the type of data available. Because no single metric can universally capture the complexity of interpatient relationships, metric selection must be purpose specific. Some metrics emphasize molecular proximity, while others are better suited to capture phenotypic or clinical resemblance. This variability underscores the need for methodological transparency and for tailoring metric selection to the specific context of the study.

This heterogeneity in metric use was mirrored in the analytical methods used. Machine and deep learning approaches were used in more than half of the studies, marking a significant methodological trend. These methods offer notable advantages for processing large-scale, high-dimensional datasets, facilitating predictive modeling and identifying complex, nonlinear patterns that may not be accessible via traditional statistical techniques. This suggests that the growing adoption of machine learning is a response to both the scale and complexity of omics data in oncology. In particular, clustering techniques, such as spectral and hierarchical clustering, were frequently used to define patient subgroups based on genetic and clinical features, supporting the hypothesis that certain molecular signatures may correlate with treatment outcomes [24-39]. Despite their strengths, machine learning methods often lack interpretability, which remains a significant challenge in their application to clinical practice. These models are also prone to overfitting, performing well on training data but failing to generalize to new cases. In addition, imbalanced test-to-training ratios in oncology datasets can introduce bias, further limiting their clinical reliability.

Network- and graph-based approaches were also prominently represented in the reviewed studies. That can be explained by the fact that these methods are especially important when modeling the relationships and interactions among biological entities and when working with data that encode complex dependencies. It was shown that techniques such as PSNs and PPI networks provide an intuitive way to represent patient similarities in the context of shared genetic or clinical features [28,29,49,53-59]. Nevertheless, network-based models are also sensitive to data weighting and metric definitions, which can significantly alter network topology and affect reproducibility. This reinforces the importance of standardizing methodological choices and validating models across diverse datasets.

The integration of different analytical approaches, particularly in hybrid models, shows promising potential for future research. For instance, combining GCNs with statistical validation techniques represents an emerging strategy for enhancing both accuracy and interpretability [53,54,61,69,70]. These hybrid approaches may offer a path forward in overcoming the limitations of individual methods while leveraging their respective strengths.

In contrast, our analysis showed that methodological choices were rarely arbitrary. Instead, they were closely aligned with research objectives: clustering was typically used for subgroup identification, deep learning and GCNs for prediction, and multimodal integration for stratification and exploratory biological analysis. These patterns suggest that patient similarity is best viewed not as a universal measurement but as a flexible framework tailored to specific goals.

In addition, the analysis of data dimensions brought additional insight by highlighting that transcriptomic, clinical, and genetic and genomic data are the most frequently used for assessing patient similarity [182]. The high use of transcriptomic data can be attributed to its direct relevance in understanding gene expression patterns that characterize cancer heterogeneity and its abundance. The integration of multiple data types, such as combining transcriptomic and clinical data, emerged as a significant trend, reflecting the need for comprehensive data to fully capture patient similarity.

Finally, the frequent study of breast, lung, and brain cancers in the context of patient similarity underscores the prevalence of these cancers and the availability of well-characterized datasets [25,66,94,175]. However, this focus also points to gaps in the study of less common cancers, highlighting opportunities for future research to explore underrepresented cancer types.

### Comparison With Previous Work

In comparison with previous literature, this scoping review provides a substantial advancement in the conceptual and methodological understanding of patient similarity by focusing specifically on its application in oncology. Earlier reviews, such as the one by Sharafoddini et al [183], primarily examined patient similarity in the context of prediction models based on electronic health record data across various medical domains. Their focus remained largely on structured clinical features and statistical similarity metrics, without a disease-specific emphasis. Similarly, the systematic review by Parimbelli et al [1] explored patient similarity within the broader framework of precision medicine, outlining its relevance to predictive modeling and treatment stratification, though without a dedicated focus on oncology.

In contrast, this review focuses exclusively on cancer research and provides a multidimensional synthesis that integrates biological, methodological, and clinical perspectives. This disease-specific emphasis enables a more detailed analysis of how patient similarity is conceptualized and operationalized in the context of tumor heterogeneity and precision oncology.

A major point of distinction lies in the methodological approaches reported. While previous studies largely discussed traditional distance-based similarity metrics and simple clustering algorithms, our review highlights the widespread adoption of more advanced computational techniques. These include, among others, GCN, SNF, and federated learning models, which allow the integration of heterogeneous data types while preserving patient privacy and ensuring model robustness. This evolution reflects the increasing sophistication of oncology research and the growing need for methods capable of handling high-dimensional multiomics datasets [15,52,53,165].

Moreover, our review extends prior work by emphasizing the interdependence between data types and methodological frameworks. Although previous literature focused primarily on structured clinical or demographic data, the findings here underscore the central role of transcriptomic, genomic, and multiomics data. These data types are often analyzed using machine learning and network-based approaches to uncover complex biological patterns [49,55].

Importantly, while earlier reviews identified patient similarity as a promising concept, they mostly discussed its potential applications. In contrast, our findings demonstrate that patient similarity is already being actively applied in oncology for critical tasks such as cancer subtype identification [39], biomarker discovery [49], and predictive modeling of survival or treatment outcomes [55]. These applications signify a clear transition from theoretical potential, as discussed in earlier work, to real-world implementation in precision oncology.

### Study Limitations

Despite its strengths, this scoping review has several limitations.

First, it relies on published literature, meaning the findings are constrained by the scope and quality of the included studies. Potential biases such as selective reporting and variability in study quality may have influenced the results. In particular, publication bias poses a concern: studies reporting positive findings, especially those successfully identifying patient similarities, may be overrepresented, while studies with negative or inconclusive results (eg, in specific cancer types) might be underrepresented. This pattern is theoretically plausible, given the tendency to publish predominantly significant findings [1,183]. Although we used a comprehensive search strategy across multiple databases, we did not include gray literature, which may further limit the breadth of the included evidence. Consequently, the inherent biases of published data remain a challenge. Future research should aim to incorporate unpublished studies and negative findings to provide a more balanced and comprehensive perspective.

Second, we limited the temporal scope to research published within the last 25 years, which could have led to the omission of earlier foundational studies.

Third, the focus on common cancers (eg, breast, lung, and brain) limits the generalizability of the findings to rarer cancers, where patient similarity remains underexplored [39,49,53,101]. In addition, synthesizing findings across studies using different definitions and metrics of patient similarity was challenging. The lack of a universally accepted definition led to inconsistencies in how similarity was measured, ranging from clinical characteristics to molecular profiles. This heterogeneity introduced biases and limited generalizability. In particular, studies relying on different data types, such as clinical records versus genomic data, varied in their ability to make strong claims about patient similarity. Consequently, findings from certain methodological approaches may carry more weight than others, introducing an inherent imbalance in the available evidence. Furthermore, the variability in methodologies, from machine learning to network-based and statistical approaches, hinders standardized clinical recommendations [24,25,28,46,182].

Finally, while the review emphasizes the importance of integrating multiomics and clinical data, it does not delve into practical guidance for addressing challenges such as data heterogeneity and interoperability, as this was beyond its scope. However, emerging solutions, such as data harmonization efforts and federated learning approaches, are increasingly being explored to tackle these issues, offering avenues for future research and application [52,61,69].

### Conclusions

This scoping review provides a comprehensive overview of the current understanding of the concept of patient similarity in cancer research and treatment.

The findings indicate that patient similarity is best conceptualized as a relational construct, reflecting the specific objectives, data constraints, and clinical priorities of each study. Rather than representing a single metric, it constitutes a multidimensional modeling paradigm, shaped by research goals, data architecture, cancer-specific contexts, and methodological design. Its successful application hinges on purpose-driven model development, rigorous validation, and the thoughtful integration of heterogeneous data sources.

To further advance the field, future research must emphasize standardization, including the development of clear and consistent criteria for defining, measuring, and evaluating similarity. In parallel, efforts should focus on expanding available data diversity and designing models that are both biologically grounded and clinically interpretable. These advancements will support the broader clinical integration of patient similarity and catalyze progress toward personalized oncology, enabling more precise, effective, and individualized cancer treatments that reflect the unique characteristics of each patient and improve outcomes across diverse populations.

## Appendix

Multimedia Appendix 1 Extracted data from all the included papers.

Multimedia Appendix 2 PRISMA-ScR (Preferred Reporting Items for Systematic Reviews and Meta-Analyses Extension for Scoping Reviews) checklist.

Multimedia Appendix 3 Details of the exclusion decisions and conflict resolution.

Multimedia Appendix 4 Distribution of the detected data.

Multimedia Appendix 5 Key research objectives that are frequently applied to define similarity in patients with cancer.

## Abbreviations

ANF: affinity network fusion
BRCA: breast invasive carcinoma
DeepLIFT: deep learning important features
GBM: glioblastoma multiforme
GCN: graph convolutional network
LUSC: lung squamous cell carcinoma
NMF: nonnegative matrix factorization
NMTF: nonnegative matrix trifactorization
PPI: protein-protein interaction
PRISMA: Preferred Reporting Items for Systematic Reviews and Meta-Analyses
PRISMA-ScR: Preferred Reporting Items for Systematic Reviews and Meta-Analyses Extension for Scoping Reviews
PSN: patient similarity network
SNF: similarity network fusion
WGCNA: weighted gene coexpression network analysis
