# Supplementary material for: Uncovering the Understanding of the Concept of Patient Similarity in Cancer Research and Treatment: Scoping Review
Source: J Med Internet Res. 2025 Aug 18;27:e71906. doi: 10.2196/71906 (PMC12402742; doi:10.2196/71906)
Supplement: Multimedia Appendix 3 [file jmir_v27i1e71906_app3.docx]

Exclusion of studies during the full-text screening phase of the review process is summarized in Textbox 1 and visualized in Figure 1.

Textbox 1. Excluded reports during full-text screening phase.

| **Non-cancer conditions**  A total of 43 studies were excluded as they focused on non-oncological conditions, making them irrelevant to this research.  **Indirect relation to the research question**  40 studies were excluded due to their indirect relation to the research questions, as they did not align closely with the core objectives.  **Background articles**  16 studies were excluded as they primarily provided background information and lacked sufficient data for core analysis.  **Language**  2 studies were excluded because they were written in languages that did not meet the inclusion criteria of this research.  **Duplicates**  2 duplicate studies were excluded, as they were not automatically identified during the initial screening. |
| --- |

Figure 1. Visualization of excluded reports during full-text screening phase.


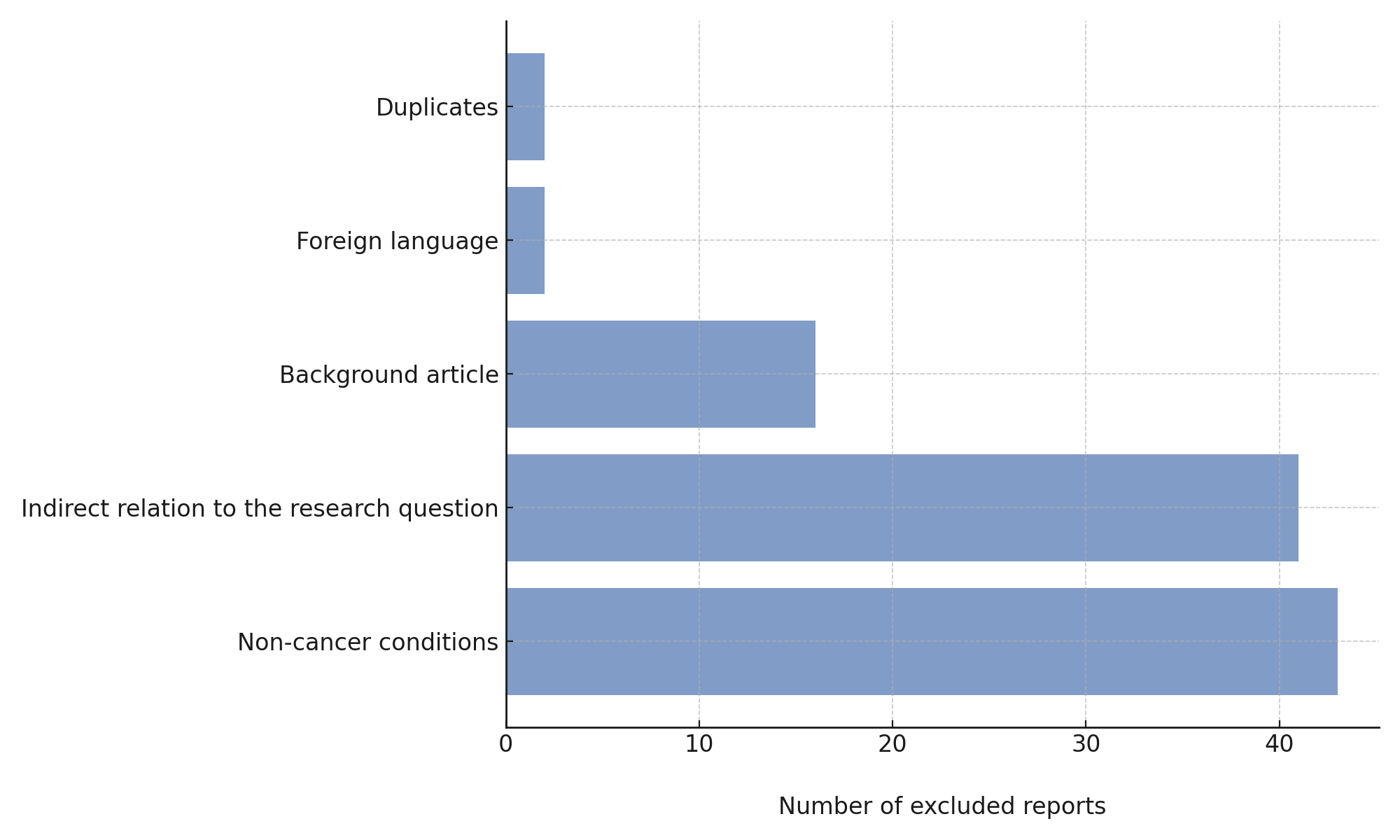


**Conflict resolution process**

Additionally, 151 records (12.8%, 151/1183) underwent conflict resolution following the Title-Abstract Screening phase, to ensure consistent application of inclusion criteria and enhancing the reliability of the selection outcomes. During  the Full-Text Screening phase, 6 conflicts (2.3%, 6/258) required resolution, leading to 2 additional papers (0.8%, 2/258) passing the screening and being included in the final dataset. These measures were implemented to ensure a rigorous and transparent study selection process, contributing to the reliability and validity of the final dataset.
