# Supplementary figures and images for: Uncovering the Understanding of the Concept of Patient Similarity in Cancer Research and Treatment: Scoping Review
Source: J Med Internet Res. 2025 Aug 18;27:e71906. doi: 10.2196/71906 (PMC12402742; doi:10.2196/71906)

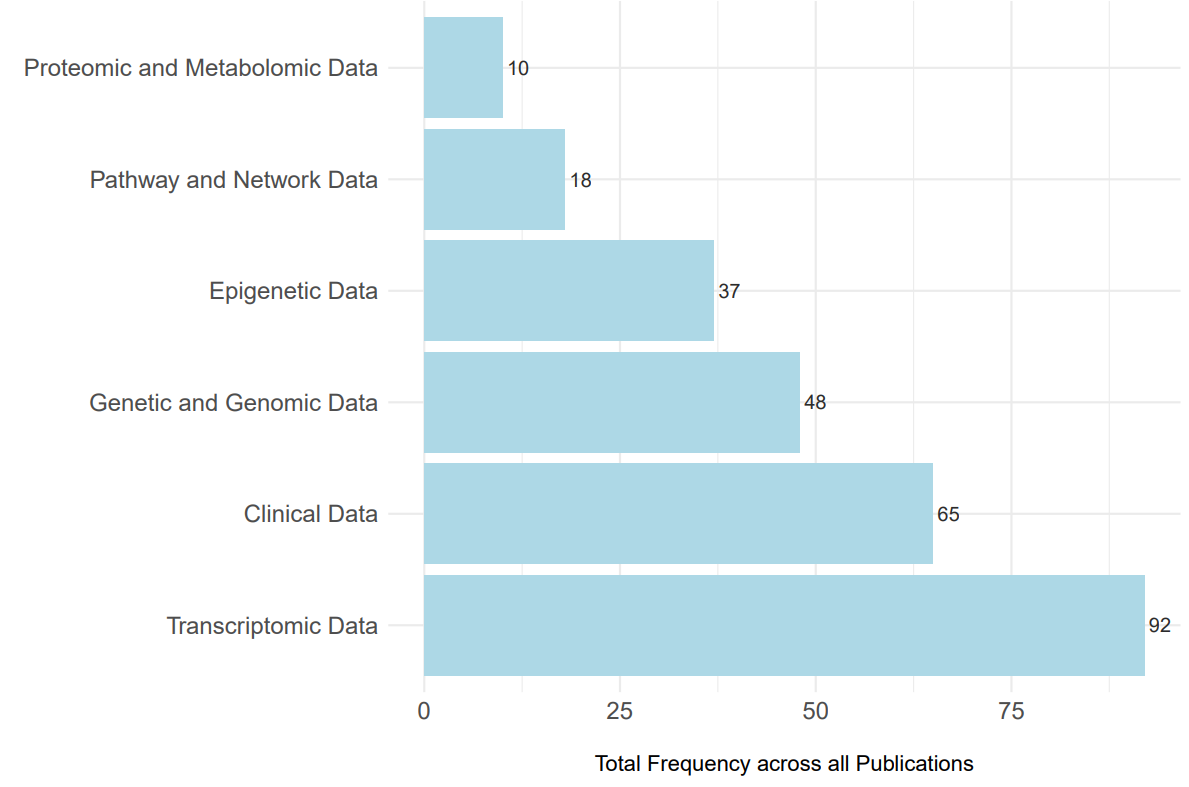

Supplement: Multimedia Appendix 4 [file jmir_v27i1e71906_app4.docx]

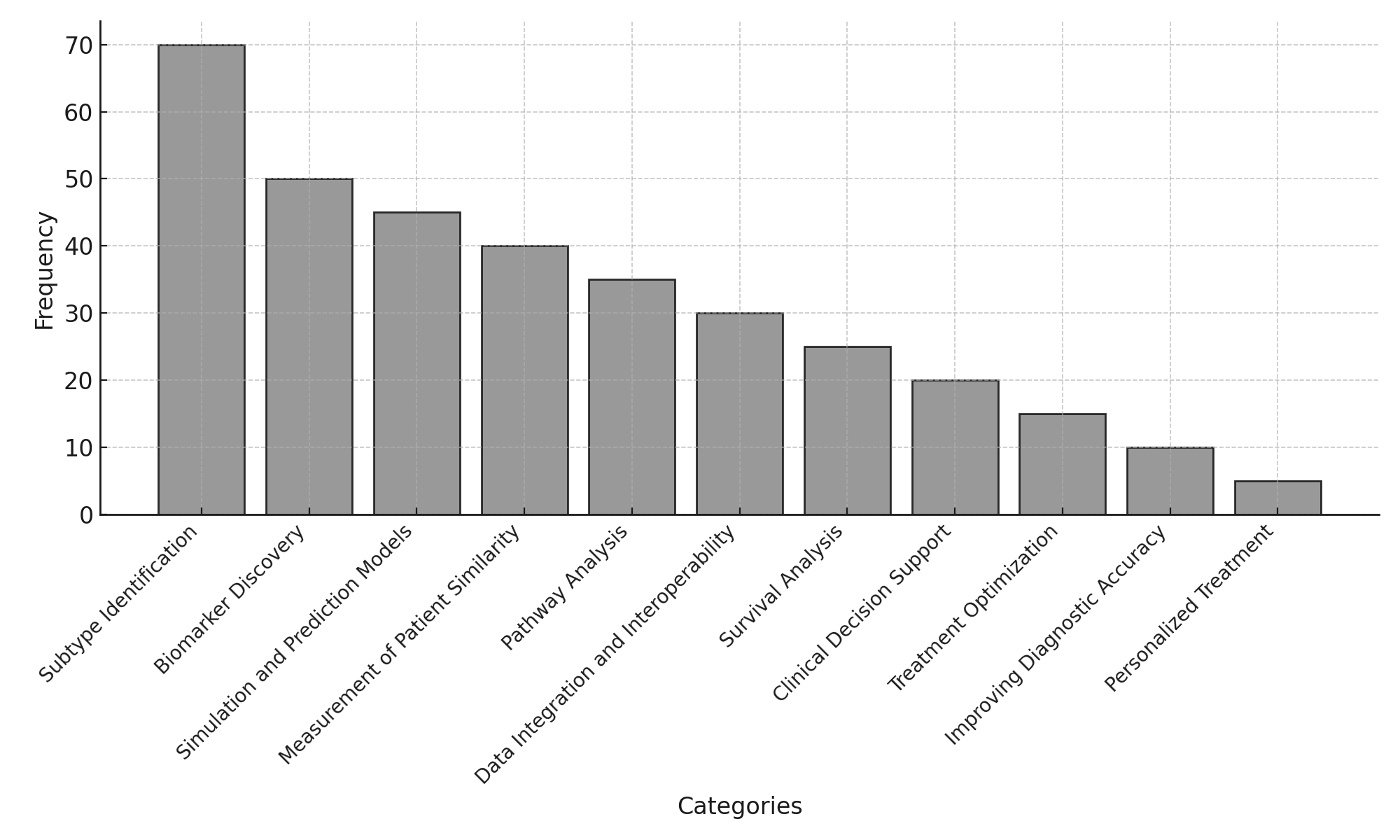

Supplement: Multimedia Appendix 5 [file jmir_v27i1e71906_app5.docx]
